# Climate-sensitive health priorities in Nunatsiavut, Canada

**DOI:** 10.1186/s12889-015-1874-3

**Published:** 2015-07-02

**Authors:** Sherilee L. Harper, Victoria L. Edge, James Ford, Ashlee Cunsolo Willox, Michele Wood, IHACC Research Team, RICG, Scott A. McEwen

**Affiliations:** 1 grid.34429.380000000419368198Department of Population Medicine, University of Guelph, Guelph, ON Canada; 2 Indigenous Health Adaptation to Climate Change Research Team: Lea Berrang-Ford, Cesar Carcamo, Alejandro Llanos, Shuaib Lwasa, Didacus Bambaiha Namanya, Montreal, Canada; 3 grid.14709.3b0000000419368649Department of Geography, McGill University, Montreal, QC Canada; 4 grid.253649.f0000000121518595Department of Nursing, Cross-Appointed with Indigenous Studies, Cape Breton University, Sydney, NS Canada; 5 Department of Health and Social Development, Nunatsiavut Government, Goose Bay, Labrador, Canada; 6 Rigolet Inuit Community Government, Rigolet, Labrador, Canada

**Keywords:** Canada, Climate change, Health, Inuit, Nunatsiavut, EcoHealth

## Abstract

**Background:**

This exploratory study used participatory methods to identify, characterize, and rank climate-sensitive health priorities in Nunatsiavut, Labrador, Canada.

**Methods:**

A mixed method study design was used and involved collecting both qualitative and quantitative data at regional, community, and individual levels. In-depth interviews with regional health representatives were conducted throughout Nunatsiavut (*n* = 11). In addition, three PhotoVoice workshops were held with Rigolet community members (*n* = 11), where participants took photos of areas, items, or concepts that expressed how climate change is impacting their health. The workshop groups shared their photographs, discussed the stories and messages behind them, and then grouped photos into re-occurring themes. Two community surveys were administered in Rigolet to capture data on observed climatic and environmental changes in the area, and perceived impacts on health, wellbeing, and lifestyles (*n* = 187).

**Results:**

Climate-sensitive health pathways were described in terms of inter-relationships between environmental and social determinants of Inuit health. The climate-sensitive health priorities for the region included food security, water security, mental health and wellbeing, new hazards and safety concerns, and health services and delivery.

**Conclusions:**

The results highlight several climate-sensitive health priorities that are specific to the Nunatsiavut region, and suggest approaching health research and adaptation planning from an EcoHealth perspective.

## Background

Changes in climate and the resultant environmental alterations continue to be documented globally [1–3]. The consequences of these changes are broad and wide-ranging, and include direct and indirect impacts on health and wellbeing [4, 5]. While the health impacts of climate change are expected to be felt globally, some populations have been identified as particularly vulnerable to health-related climate change impacts, including Indigenous peoples and communities in Canada [6–12], Australia [13, 14], Uganda [15], Peru [16], Greenland [17], and United States [18–20]. For instance, in the Circumpolar North, the rapidly increasing atmospheric temperatures have drastic impacts on ice coverage, water systems, permafrost, flora, and fauna, all of which have important implications for the health and wellbeing of Inuit inhabiting the North [6–11, 20].

The impacts of climate change on health are often localized and dependent on geographical location, biophysical factors, social and environmental relationships, cultural practices, and traditional knowledge [10, 21]. Indeed, in the climate-health literature, vulnerability to climate change at the local scale can be defined as a function of “*exposure to climate-related health risks, sensitivity to these risks, and adaptive capacity to address, plan for, or manage them*” (Ford 2012 [10], pg. 1260). Local vulnerability assessments underpin efforts to prepare for and adapt to current and future climate change impacts on Inuit health [10, 12, 22–24]. While many frameworks are available to guide assessments of human health vulnerability to climate change, all share a common first step: to work with local populations and regional stakeholders to identify and describe the most important climate-sensitive health outcomes (i.e. health outcomes that are sensitive to climate change and variability) for a given population/region [22, 23, 25–31]. This step is necessary to identify and understand climate-sensitive health priorities at individual, household, community, and regional scales to guide health research and effectively inform the development of public health adaptation strategies and interventions [10, 22–24].

Responding to the need to work collaboratively with communities to examine health priorities, this study piloted a participatory process to characterize the climate-sensitive health priorities in Nunatsiavut, Labrador, Canada. The objectives of the study were to: (1) develop a baseline understanding of the climate-sensitive health outcomes currently affecting Labrador Inuit; (2) identify climate-sensitive health issues that are anticipated to affect Labrador Inuit in the future; and (3) prioritize climate-sensitive health issues to inform future research and adaptation strategy and policy development in the Nunatsiavut region.

## Methods

### Nunatsiavut, Canada

**Fig. 1:**
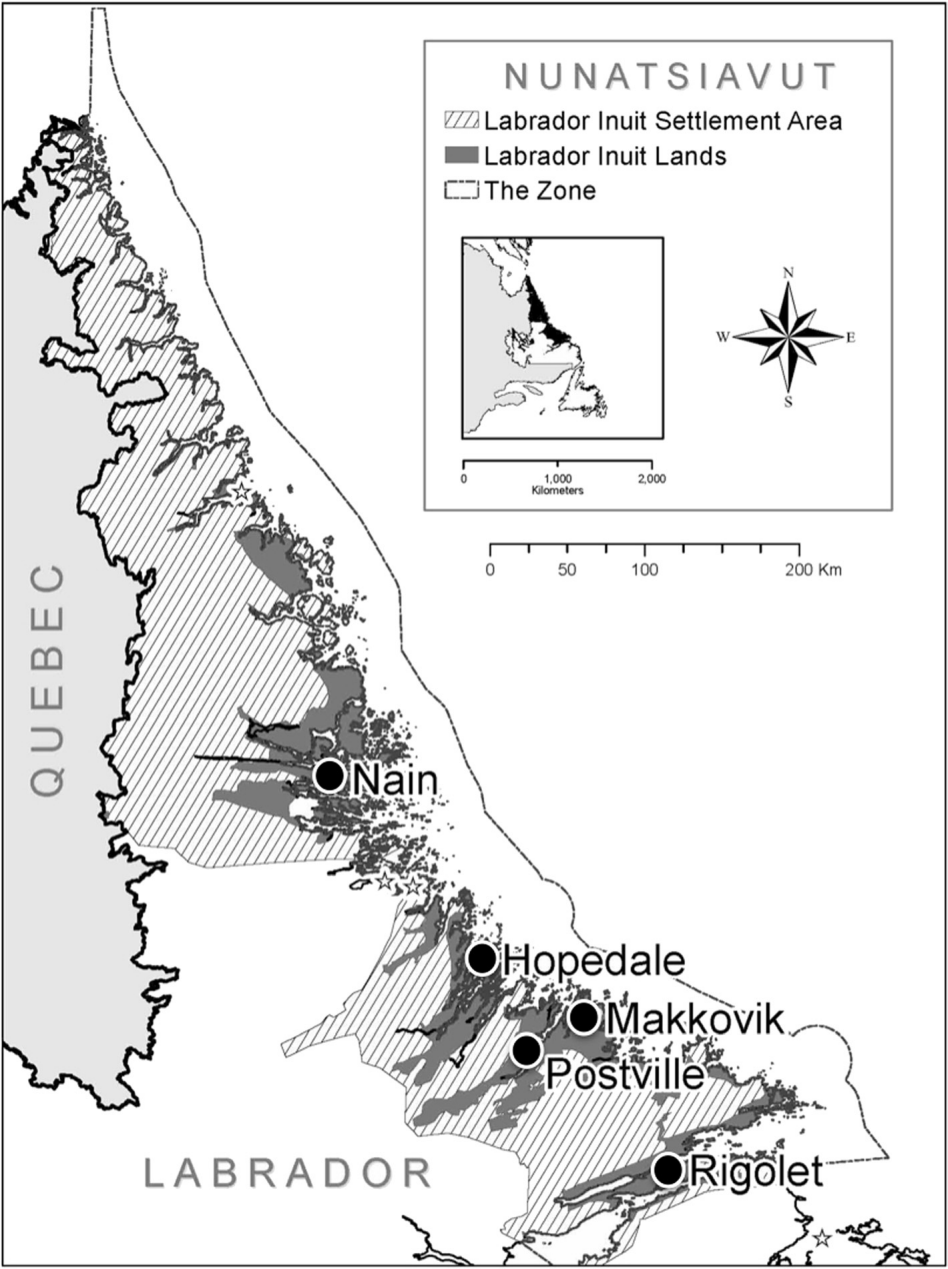
A map of the five Inuit communities within the Labrador Inuit Settlement Area in Nunatsiavut, Labrador, Canada

Inuit are one of the three constitutionally-recognized Indigenous groups in Canada, along with First Nations and Metis/Métis. There are approximately 55,000 Inuit living throughout Canada, primarily within the four settled Inuit land claim regions of Inuvialuit, Nunavut, Nunavik, and Nunatsiavut. Nunatsiavut (Fig. 1) is located on the Northeast coast of Labrador, and contains five communities within the 72,520 km^2^ land claim settlement area (from north to south): Nain, Hopedale, Postville, Makkovik, and Rigolet. All five communities are remote and only accessible by a commercial year-round plane service and a seasonal ferry service. To access cabins, traditional lands, and hunting grounds outside of the communities, residents travel by snowmobile over snow and ice in winter months, and over water in small personal boats in the summer months. Labrador Inuit continue to rely on the local ecosystem for livelihoods, cultural continuity, and wellbeing, and depend on hunting and gathering of caribou, seals, ducks, geese, eggs, fish, and berries for partial subsistence [6–8].

### Healthcare provision in Nunatsiavut

Primary healthcare, pharmaceutical, and clinical mental health and addictions services are provided by the provincial government through the Labrador-Grenfell Health Authority, which manages community clinics, staffed with at least one nurse, and a physician who visits each community on a rotating basis. Residents travel by airplane to southern health centres for childbirth, some mental health counselling, addictions rehabilitation, medical emergencies, and for nearly all appointments with specialists or physicians that require diagnostic equipment. Public health, mental health, and some counselling and addiction services are provided by the regional Inuit government, the Nunatsiavut Government (Department of Health and Social Development), which has a Community Health Team in each community. The Nunatsiavut Government also administers the Non-Insured Health Benefits program for Nunatsiavut beneficiaries.

### Research design

A concurrent mixed-methods research design was used, whereby qualitative and quantitative data at regional and local scales were collected and analysed concurrently and then descriptively combined and compared for convergent and divergent ideas and concepts [32–34]. This research was a part of a larger international initiative called the Indigenous Health Adaptation to Climate Change project (www.ihacc.ca), with the work reported here conducted to inform priorities for this research program as per principles of community-based participatory research [7, 15, 35–37]. Written informed consent was obtained from all participants and the research protocol was reviewed and approved by the Nunatsiavut Government Research Advisory Committee and the Research Ethics Boards at the University of Guelph and McGill University.

### Regional data collection: interviews with government employees

In-depth interviews with government employees working within the Nunatsiavut region were conducted (*n* = 11) in 2010, totalling 604 minutes of recorded discussion (Table 1); the average interview lasted 50 minutes (range: 34–79 minutes). The semi-structured interview guide included open-ended questions aimed to identify and characterize health priorities and vulnerability to climate stressors in the Nunatsiavut region. The interview guide promoted a conversational interview format [38] and was pre-tested for content and context by academics and health practitioners. Individuals representing provincial, regional, and community levels were purposefully selected based on their job description and work experience, invited (via email) for a private and confidential interview, and interviewed in person at each participant’s workplace. Then, all participants were invited to attend a focus group discussion to examine and verify the preliminary findings.

**Table 1:** Demographic information of government interviewees, community survey participants, and PhotoVoice participants in Rigolet, Nunatsiavut

Demographic Information	Government	PhotoVoice	Community Survey Participants (%)	
	Interviewees (%)	Participants (%)	CCI survey^a^	BPS survey^b^
	n=11	n = 11	n = 75	n = 112
Age				
Youth (0–20)	0 (0)	1 (9)	4 (5)	8 (7)
Adult (21–50)	9 (82)	7 (64)	43 (57)	69 (62)
Elder (Over 50)	2 (18)	3 (27)	28 (37)	30 (27)
No response				5 (4)
Sex				
Male	2 (18)	6 (55)	44 (59)	52 (46)
Female	9 (82)	5 (45)	31 (41)	60 (54)

^a^Climate change impacts (CCI) on health survey (‘CCI survey’): Collected data on the observations, beliefs, and attitudes about climate change impacts on health

^b^Bio-psycho-social (BPS) impacts of climate change survey (‘BPS survey’): Collected data on the perceived bio-psycho-social impacts of climate change

### Community data collection: PhotoVoice workshops and surveys

To provide community-level and individual-level perspectives to the research and contextualize the insights provided by regional stakeholders, PhotoVoice workshops and two community surveys were conducted in Rigolet, Nunatsiavut.

### PhotoVoice workshops

**Fig. 2:**
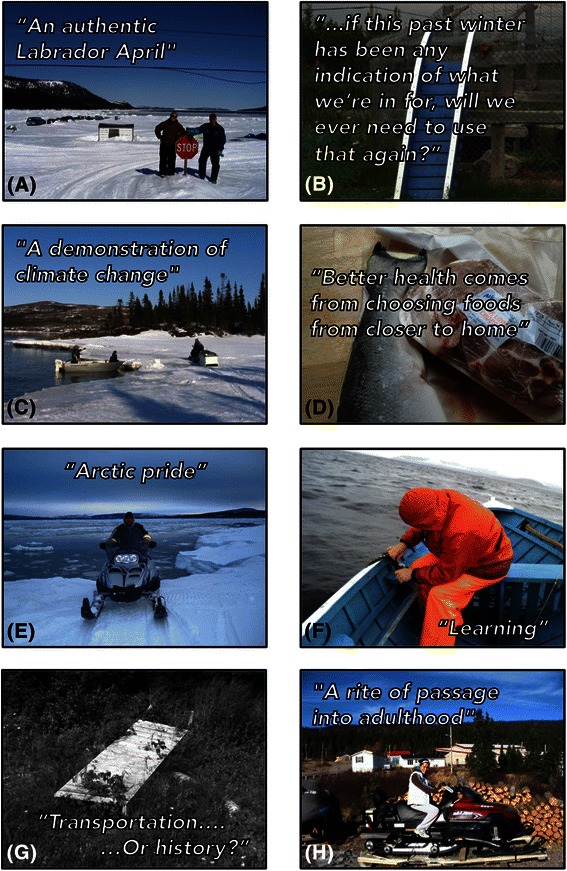
Key photos and quotes/messages (**a**-**h**) selected by PhotoVoice participants in Rigolet, Nunatsiavut, Labrador, Canada in 2010

PhotoVoice was used as a community-based participatory data collection technique, providing the opportunity for community members to take photos of areas, items, or concepts that expressed how climate change impacts their health. PhotoVoice is an interactive method that engages participants to take new photographs or gather already-taken photographs that reflect their ideas, thoughts, and feelings on a particular subject. These photos then become the foundation for group discussion and dialogue around the emergent themes, as well as for knowledge sharing beyond the group. This technique is considered to be a culturally-appropriate method for community-based participatory research in Indigenous settings [39], and has been used in other Inuit communities [40, 41]. Two PhotoVoice (PV) workshops with all female participants (*n* = 5) and one workshop with all male participants (*n* = 6) were conducted in 2010 (Table 1), with a total of 422 minutes of recorded discussion and 36 photos selected by participants (Fig. 2). The PV workshops were co-facilitated and involved five steps: (1) PV participants discussed climate change and health using concept-mapping techniques to facilitate conversation [42]; (2) Over a 2-day period, PV participants took new photographs with digital cameras, as well as collected old photographs that related to the workshop topic; (3) The groups re-convened and (a) selected which photographs to share with the group, (b) explained the stories and messages behind their photos, and (c) collectively grouped the photos into common, re-occurring themes; (4) The community facilitators assisted PV participants through a reflective process, with numerous one-on-one follow-up consultations to select quotes or messages to accompany each photo; (5) PV participants decided how they wanted their photos used, and identified creating a photo-bank on the town website, printing a PhotoVoice book, and using photos in publications for researchers and policy makers.

### Community surveys

Two community surveys were conducted in Rigolet, Nunatsiavut to understand community-level trends and perceptions of climate-sensitive health priorities. Information about the survey development and administration are described elsewhere in detail [6, 7]; briefly, a member from every household who was in the community during the sampling period was invited to complete a short questionnaire that was administered by trained community personnel. The first survey (2009) collected data on the observations, beliefs, and attitudes about climate change impacts (CCI) on health (‘CCI survey’); and the second survey (2010) collected data on perceived bio-psycho-social (BPS) impacts of climate change (‘BPS survey’) [6]. Of the 100 people available in the community during the study period to participate in the CCI survey, 75 participated (response rate = 75 %), and of the 117 people available in the community to participate in the BPS survey, 112 participated (response rate = 96 %) (Table 1).

### Analysis

#### Qualitative analysis

All interviews and group discussions were recorded with permission, transcribed verbatim by a professional company, and hand-checked for accuracy. A systematic and iterative qualitative data analysis approach [43, 44] was used that included five iterative steps: data familiarization; identification of theory-driven/deductive and data-driven/inductive codes [45]; identification of themes by writing reflective memos and creating concept/thematic maps [46]; development of a codebook [47] and coding the transcripts by labeling the text; and, finally, review and discussion with the community research team and research participants to ensure accuracy, reliability, and authenticity of the analysis. Qualitative data analysis software (Atlas.ti, version 6) was used to assist in data organization and retrieval [43].

#### Quantitative analysis

The quantitative survey data for the CCI survey and BPS survey were analysed separately. The data were explored using descriptive statistics, then potential differences in climatic and environmental change observations (outcome variables) between age groups, gender, and perceived impacts on health, wellbeing, and lifestyle (predictor variables) were examined using univariable exact logistic regression using exact conditional score tests. Excel (version 14.3.6) was used to manage data and create graphs, and all statistical analyses were conducted using Stata IC (version 11.2).

## Results and discussion

### Observed climatic and environmental change: “It is meant to be cold, and snowy, and icy, and crisp, and fresh, and bright here”

Most participants noted changes in climate and increased climate variability based on personal observations over their lifetimes, or from information shared by Inuit Elders and seniors. Nearly all community survey respondents reported changes in temperature, snow, ice, rain, or weather patterns over their lifetime (CCI survey: 74/75 (98.7 %); BPS survey: 107/108 (99.1 %)). PhotoVoice (PV) participants shared several photos demonstrating decreased ice and snow over their lifetimes (Fig. 2a, c). PV participants noted a general increase in year-round temperature, with hotter summers and milder winters, resulting in reduced ice coverage and thickness, and more rain and less snow precipitation than in living memory of the participants. These climatic changes and variability were described as “wrong,” “very unusual,” “unheard of,” “wicked,” “sudden,” “really obvious,” “really startling,” “quite profound,” “uncomfortable,” “very strange,” “worse,” “treacherous,” “horrible,” “different,” “big,” “really bad,” “depressing,” and “severe.”

Weather and climate variability (i.e. fluctuations in weather from day to day, or year to year) was described by several interviewees, including observations of less predictable and more extreme weather, unusual seasonal timing, as well as changes in the timing, frequency and duration of storms. This increased climate variability was described as “bizarre,” “odd,” “strange,” “huge,” “not normal,” “unpredictable,” “variable,” “not predictable,” “not stable,” and “episodic.” The weather in each season was called “questionable,” “less predictable,” “untrustworthy,” “a totally different season,” and “definitely different than what was expected here normally.”

The changes in climate and weather variability observed by participants are consistent with previous research in the Canadian North documenting local observations of changing climatic conditions [1, 2, 11, 48], and the resultant environmental changes observed by participants supports findings from past research [11, 49, 50]. For instance, the changes in ice conditions observed by participants reflects a documented 73 % reduction in maximum sea ice coverage in the Northern Labrador Sea over the past 50 years at a rate of 17 % (1,536 km^2^) per decade, which is the fastest rate of reduction and the greatest cumulative reduction in sea ice coverage in Canada [51].

### The Land, Health, and Wellbeing: The land “is in their bones and in their blood”

Health and wellbeing were described by participants as inextricably “interlinked” to “the land” (the environment). Interview and PV participants described the Inuit relationship to the land as “powerful,” “amazing,” “a need,” “a tradition,” “not an option,” “expected,” “a given,” “sheer want and internal desire,” “perfect,” “a sense of being,” and that “nothing can replace it.” Participants described that the land is “intertwined and interconnected” to various determinants of Inuit health. This understanding of health reflects the Inuit ecocentric concept of self, in which “the individual is in constant transaction with the physical environment” and “gives a central role to connections among individuals and to place in the health and well-being of the person” (Kirmayer et al. [52], pg. 292). In the public health literature, the environment is considered one of fourteen social determinants of health [53] and is often viewed as the most neglected or overlooked of these determinants [54, 55]. In Nunatsiavut, however, participants did not view the environment as a separate determinant of health, but rather viewed the environment or “the land” as interconnected to—and indeed supporting and underlying—each determinant of health. Health was viewed as an outcome of complex relationships between the environment and other determinants of health, in that the environment influences and is influenced by determinants of health; such concepts of health have been supported among Indigenous populations in multiple contexts [56]. As one Inuit interviewee explained,

I guess it's all the pieces, like dominoes, all touches each other. I mean everything you do, [our] Inuit way of life and our way of thinking is all intertwined and interconnected [to the environment]. So, something as significant as changes in the temperature, and in snow and rain and that kind of thing, it’s all going to have a ripple effect.

Another Inuit government interviewee explained,

In regions where you have cultures like the Inuit, they need to think, you know everybody should be thinking more about looking at things through the social determinants [of health], because if you don't, you're never going to fix them. You know, you have to look at the causes of the causes. And climate change is a huge, huge impact for the Inuit.

Participants believed that the close link between the land and health increases sensitivity to climatic change related impacts on health. Indeed, in the community surveys, most respondents believed that the observed climatic and resultant environmental changes were impacting lifestyles or health (CCI survey: 68/75 (90.7 %); BPS survey: 65/110, (59.1 %)). Government interviewees and PV participants discussed the “huge implications” for these climatic and weather changes, including reduced snow and ice acting as additional barriers to accessing the land to visit cabins and to hunt, fish, trap, and harvest food—activities which are extremely important in Nunatsiavut and across the North and have documented positive influence on mental well-being [6, 8, 57]. As one interviewee explained,

I don’t see anything positive about it [climatic change] here. It is meant to be cold, and snowy, and icy, and crisp, and fresh, and bright here. For the most part, that’s what is natural and normal here. And that is what people expect and love about this weather here, that is why [Inuit] stay here–it is in their bones and in their blood.

This finding is supported by previous research in Nunatsiavut [6, 8, 9, 11], as well as other research in other Circumpolar communities in Canada [40, 58, 59], Greenland [17], Norway [60], United States [18, 20], and Russia [61].

### Pathways of climate-health impacts

**Fig. 3:**
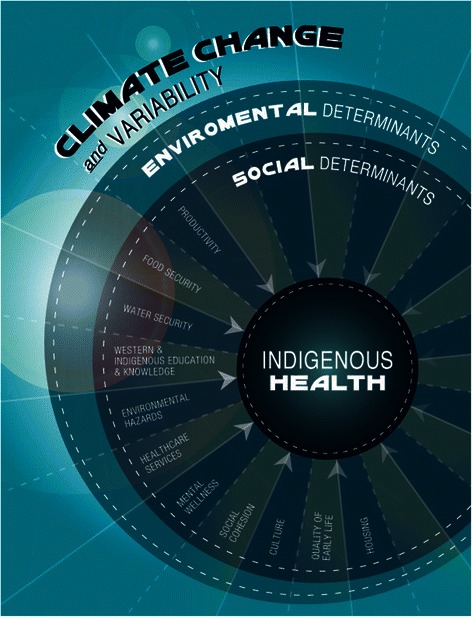
The pathways through which climate change impacts on Indigenous health in Nunatsiavut, Canada based on participant interviews, focus group discussions, photovoice workshops, and the broader literature

Participants believed that these relationships and interconnections between the land and health increased the region’s sensitivity to climate change impacts on health. Participants emphasized the importance of interactions between and among the environment and culture, environmental hazards, food security, water security, productivity, mental wellness, social cohesion, Western and Indigenous education and knowledge, housing, and healthcare services (each of these themes are described in detail below; Fig. 3).

#### Culture and health: “Culture as a healer”

**Fig. 4:**
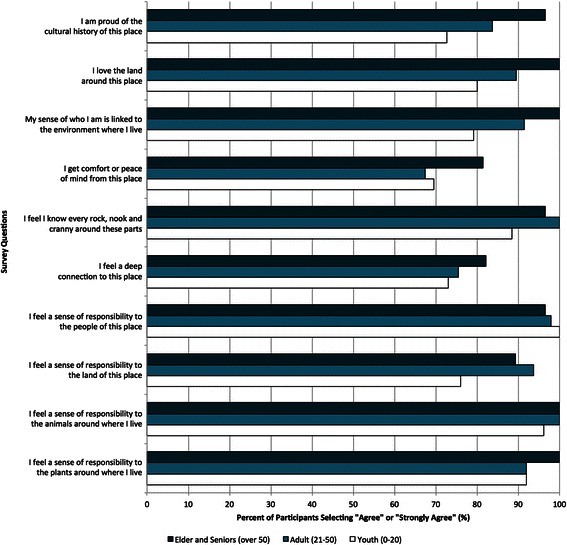
Percentage of bio-psycho-social (BPS) survey participant’s responding “Agree” or “Strongly Agree” to questions about connections to the land, people, and animals by age (youth n = 8; adult n = 69; Elder n = 30) in Rigolet, Nunatsiavut in 2010

Culture was clearly linked to the land by all participants, because the Inuit relationship with “the land” underpins several Inuit values and forms the foundation of Inuit identity (Fig. 2b, g, h, and 4), which is supported by previous research in the region [6]. Participants described how observed climatic changes were impacting cultural practices, especially in recent years. For instance, in the CCI survey, the odds of reporting climatic change impacts on lifestyle was higher for those observing changes in snow, water systems, and wildlife (Table 2), which could suggest that changes in snow, ice, water systems, and wildlife has had important and observable impacts on Inuit lifestyles. Furthermore, reporting climatic change impacts on lifestyle significantly increased the odds of ranking climatic change as an important issue for the community (Table 2), indicating that the climate-related disruptions to lifestyle are linked to community concern about climatic changes. Interview and PV participants reported that increased temperatures and rainfall was resulting in less snow coverage, as well as unstable and unpredictable ice conditions, which can impact the ability of Inuit to practice land-based activities. The impacts of changing climatic conditions on the ability to engage in land-based activities has also been documented by research across the North [6, 8, 21, 24, 58, 59, 62, 63]. As one interviewee explained, “the environment supports the culture that was strong and in order to get the culture stronger, we still need that [cold] weather to do that.”

**Table 2:** Results from the univariable exact logistic regression models based on survey data from Rigolet, Nunatsiavut, Canada

	n	Odds Ratio		p	95 %
		CCI survey^a^	BPS survey^b^		Confidence Interval
Reported climatic change impacts on lifestyle					
Did not observe changes in snow	12	Ref.			
Observed changes in snow	63	6.2		0.018	1.30-40.40
Did not observe changes in water systems	28	Ref.			
Observed changes in water systems	47	3.8		0.016	1.25-12.12
Did not observe changes in wildlife	34	Ref.			
Observed changes in wildlife	41	3.5		0.024	1.16-11.10
Did not rank climatic change as an important issue for the community	13	Ref.			
Ranked climatic change as an important issue for the community	54	7.8		0.004	1.79-41.92
Reported climatic change impacts on health					
Did not report changes in wildlife or vegetation	34	Ref.			
Reported changes in wildlife or vegetation	41	5.4		0.026	1.78-34.57
Did not observe changes in the quality or quantity of fresh water	28	Ref.			
Observed changes in the quality or quantity of fresh water	47	8.9		0.003	1.88-58.00
Rated changes in fresh water quantity as a “strong threat” or “extreme threat”					
Did not report concern that climatic change was causing illness	70		Ref.		
Reported concern that climatic change was causing illness	31		2.8	0.039	1.04-7.87
Rated changes in fresh water quantity as a “strong threat” or “extreme threat”					
Men	50		Ref.		
Women	60		6.0	0.003	1.69-27.55
Rated changes in fresh water quantity as a “strong threat” or “extreme threat”					
Men	50		Ref.		
Women	60		2.7	0.026	1.11-6.67
Reported feeling angry from changes in the environment					
Men	50		Ref.		
Women	60		2.8	0.028	1.10-7.53
Reported feeling scared from changes in the environment					
Men	50		Ref.		
Women	60		3.0	0.014	1.23-7.80
Reported feeling frustrated from changes in the environment					
Men	50		Ref.		
Women	60		2.8	0.018	1.17-6.84

^a^Climate change impacts (CCI) on health survey (‘CCI survey’): Collected data on the observations, beliefs, and attitudes about climate change impacts on health

^b^Bio-psycho-social (BPS) impacts of climate change survey (‘BPS survey’): Collected data on the perceived bio-psycho-social impacts of climate change

Land-based, cultural activities are an important component of physical activity for many Inuit [64, 65]. While previous research suggests that self-reported physical activity is higher for Inuit compared to the national average [66, 67], PV participants reported a recent decrease in physical activity in the community. Participants identified several reasons for this trend, and changes in climate and weather were identified as contributing factors. For example, PV participants linked changing weather patterns and an “unpredictable climate” to decreased land access, hunting, trapping, and gathering, which “impacts how [physically] active you are.” A decrease in physical activity was perceived to contribute to current and possible future increases in “diabetes,” “obesity,” “heart disease,” and “even some cancers related to obesity.” As such, participants identified “fitness,” “sedentary lifestyles,” “participACTion,” the ability to “do land-based activities,” “physical activity” and “activity and encouraging people to be active in their community–whether they are on the land or just walking around town” as climate-sensitive health priorities.

#### Environmental Hazards: “Jeopardizing their lives going out on unstable elements”

Hazards on the land and increased injury was another example participants used to illustrate how climatic changes were one of several factors (e.g. new technology, de-skilling among youth [24, 48, 62, 68]) contributing to, or further exacerbating adverse health outcomes. Many participants described concerns about climatic change and variability creating “new” hazards on the land (or further exacerbating existing risks), and reported increased unintentional injuries, accidents, and deaths (Fig. 2c). Community members noted that recently “you really don’t know what is safe and what isn’t out there [anymore]” and consequently “the ice is not predictable, it is not stable, people don’t trust it.” A community member worried that in the future,

suddenly the places that you can go for generations in the past, you can't because the snow has melted or the ice is melting, or they get caught somewhere. You know someone might have to go and search for people that are missing because they were on skidoo and now you need a boat.

Government interviewees and PV participants reported that these changes were resulting in people “jeopardizing their lives going out on unstable elements,” “people going through the ice,” “family members that was off [on the land] and didn’t come back,” “issues with accidents,” “people being in danger,” and “that there have been near misses already.” Consequently, “safety,” “security on the land,” and “accidental death through changes in ice conditions and weather conditions” were identified as important climate-sensitive health priorities for the Nunatsiavut region. While research on unintentional injury and trauma on the land is limited in the North [69], previous research in Nain, Nunatsiavut indicated increased and new hazards while travelling due to the reduced strength, duration, and extent of sea ice, as well as a decreased ability to predict weather and ice conditions [70]. Most accidental injuries or emergencies on the land reported between 1995–2010 in Nain were due to weather or ice conditions; however, no long-term trends were apparent [70]. These findings are similar to other research in other Circumpolar regions. In Alaska, for instance, unusual weather and interrupted travel plans were significantly associated with increased self-reported accidents, hypothermia, and frostbite [20]. Other research suggests that the erosion of some traditional skills also increases community vulnerability to climatic changes being observed [48, 63, 68]. However, the link, if any, between environmental change and increased accidents on the land remains unclear and more empirical research is required to understand what factors might be associated with and contribute to increased accidents and injuries on the land.

#### Food Security: “That one, it’s almost hand-in-hand”

Interviewees explained that country food (i.e. food hunted and gathered locally, including caribou, fish, seal, ducks, eggs, berries, and so on) provided “good supplements,” had high “nutritional value,” and was “high in antioxidants” and “rich” in “nutrients”, which is reflected in the nutritional content of these foods [71, 72]. Participants explained that food security was ultimately dependent on the land; to obtain and consume country food, the environment must be able to support healthy populations of animals and vegetation (Fig. 2d). Recent changes in weather patterns were commonly reported to be impacting the availability and accessibility of country foods, which participants believed had adverse health implications. For instance, in the CCI survey, the odds of reporting climatic change impacts on health was significantly higher for those reporting changes in wildlife or vegetation (Table 2), illustrating the importance of wildlife and vegetation for health. As one Inuit government interviewee noted, “Climate change and weather, especially for food security - that one, it’s almost hand-in-hand.” PV participants explained that increased temperature and rainfall, as well as the intensity and frequency of storms were impacting the ability of Inuit to hunt, trap, fish, and gather foods. For example, “people didn't get out [at] seal hunting time like they normally would or for a length of time because the ice was just so unsafe [this year].” Furthermore, the impact of observed climatic changes on species migration, distribution, population size, and health supports scientific evidence in Nunatsiavut and in other Inuit regions. For example, the timing of recent ice reductions in Eastern Canada have coincided with peak pupping periods for harp seals, resulting in increased seal mortality and disease [73]. Other studies have found that caribou migration patterns are impacted by climatic variables, with herds preferring regions with higher snowfall in the winter and cooler areas in the summer [74]. Under climate change scenarios, it is also expected that the George River caribou herd in Labrador will move north, and decrease its range area to north-eastern Labrador [74]. Participants also were concerned about changes in vegetation, especially berries and mushrooms, which is supported in the literature [75]. As one Elder commented,

Last year we had no blueberries. And I mean blueberries are almost a staple. They're so good for you and they're so high in the antioxidants. There were none. And there is none because it got really hot in the spring and the berry, the bushes fried up. But to have no blueberries, it's unheard of. You know, to have that happen [long pause].

Participants also described how some of the observed changes in climatic conditions were contributing to an increased reliance on retail food, which was described as “sugary,” “junk,” “garbage,” “high sodium, high sugar food.” The increased rainfall and increased intensity and duration of storms were perceived to result in more interruptions in retail food delivery to the remote communities in Nunatsiavut, further illustrating the sensitivity of the food system to climate. As one government interviewee explained,

The conditions were horrible. People didn’t get what they normally get for caribou and then you rely on store food junk, because what other option do you have when you live in a remote fly-in only community? And, the foods sources that you usually get to, you can’t reach. There is not enough snow, there is not enough ice. It’s alarming that we are just seeing the beginning of climate change… And if the weather is down [bad] for five weeks, how do you get in and out of your community to access services? How do you get food in there? I mean the stores had to actually, I mean their stock was down to bare bones because there was no way to get food in.

Many participants also linked food security to other factors, including income, mental health, education, changing cultural practices, and increasing costs of supplies (e.g. fuel) and maintaining equipment (e.g. snowmobiles, boats). As such, participants believed that climate factors were putting additional pressure on an already stressed food system.

Some government interviewees also expressed concern regarding the safety of country food due to changes in temperature impacting country food preparation techniques (e.g. “botulism”) and warmer temperatures encouraging pathogen growth, which has been suggested in other Northern communities [76, 77]. Furthermore, some interviewees were concerned about the ability to adequately maintain safe temperature-holding during delayed shipment of retail foods (e.g. to prevent growth of foodborne pathogens such as *E. coli* and *Salmonella*), which has been suggested in other Inuit communities [78–80] and documented in North America [81–86].

#### Water Security: “There wasn’t a stitch of water – absolutely dry ground”

Participants identified concerns about the impacts of observed changing climatic conditions on the quantity, quality, and accessibility of safe drinking water. Community members explained that some “ponds have completely dried up.” As one community member explained,

It also makes quite a big difference too at the cabins where you generally depend to get your water source while you’re there. There’s very little water trickling on the brooks and this is due to limited snow but then it’s a lot to do with the heat as well. The heat is really drying up a lot of those all those ponds and a lot of those brook areas.

Furthermore, while several government interviewees described the current sources of drinking water in the region as “pretty pristine,” there was concern over how observed climate-related environmental changes could compromise the safety of drinking water in the future. Community members and government employees described concerns about high impact weather events, including heavy rainfall and rapid snowmelt leading to increased run-off and compromised water quality, which can adversely impact health. For instance, in the CCI survey, the odds of reporting climatic change impacts on health was higher for those observing changes in the quality or quantity of fresh water (Table 2). Furthermore, in the BPS survey, rating changes in fresh water quantity as a “strong threat” or “extreme threat” significantly increased the odds of reporting concern that climatic change was causing illness (Table 2), illustrating community concern about climatic influences on water-related health issues in the community.

More specifically, government interviewees and PV participants discussed concerns about future climatic changes leading to “higher rates of *Giardia*,” “potential for algae growth,” increased water turbidity leading to increased “chemicals that you add and all the by-products from that,” “drinking water diseases,” and “enteric diseases.” Similar concerns about climatic change impacts on drinking water safety in our study were also reported in other Nunatsiavut studies, including observed changes in water quality [6, 8, 11, 87], poor water quality [88, 89], and associations between weather events, drinking water quality, and related clinic visits [90].

#### Productivity: “An internal want to provide”

Many participants described “productivity” as an important contributor to health and wellbeing, which included paid employment (e.g. a formal job) and unpaid activities (e.g. hunting, gathering, volunteering). Productivity was often described in terms of being able to connect with the land in some regard, because it “provides you with a sense of capability and a sense of peace within yourself.” As one government interviewee explained,

They [Inuit] have a real sense of purpose when they’re there [on the land]. You know, in the past when they [Inuit] lived on the land, everybody had a role and everybody had a job to do and those kinds of things. I think people, when they're out on the land, they feel that sense of independence and interdependence… People completely change–it's like they have a different personality when they're out on the land and connected to that. And there’s a real spiritual quality about that as well.

The observed changes in climate were challenging participants’ ability to access the land, which they believed impacted peoples’ ability to feel productive in their family and community. In particular, government interviewees described that the practice of hunting and sharing food contributes to defining “self-worth” and productivity. As one interviewee described,

Horrible, horrible, horrible. So, that is my first thought [about climate change]… already look at what has happened with people losing that sense of identify and pride and people feel proud of bringing that fish home and that caribou home and they share it with people. So, losing the ability to provide that, and give that satisfaction from giving and sharing and feeding your family–it is going to continue to erode.

A community member explained that land-based “activit[ies] and community and sharing and a sense of being able to be productive with yourself comes with or creates health” (Fig. 2e). Consistent with the literature [6, 8, 9, 57], government interviewees and PV participants linked decreased productivity with poorer physical health outcomes (e.g. “fitness,” “less active,” “nutrition”) and mental wellness (e.g. “less happy,” “sense of identity,” “pride,” “sadness,” “abuse of substances,” “self-worth,” “emotional health”).

#### Mental Wellness: Being on the land “is the best and the most beautiful, peaceful, rejuvenating thing”

Government interviewees explained that, similar to other Indigenous communities, there were mental health and mental wellbeing concerns in Nunatsiavut rooted in the legacies of colonization. Interviewees also linked mental health to other factors, including lack of housing, culture, income distribution, and productivity. Government interviewees and PV participants, however, believed that regularly and safely accessing and connecting with the land was essential for mental wellness (Fig. 2e). One government health worker explained,

They [Inuit] get rejuvenated, they feel good when they are out of the land and when they are in nature, when they are [at] their cabin, when they are hunting or fishing, when this happens they feel good. So, that to me is healing–feeling good about yourself, being proud of what you are doing–feeding your family, being creative, being resilient.

Similar to previous research [57], government interviewees also discussed how a changing climate could potentially further impact, “magnify”, “build upon”, and bring “more to the surface” other sources of previous mental health trauma. As one community member described,

I think the trauma of being forced to assimilate … will be felt further if climate change affects [land-based] activity so you don’t find that worth through [land-based] activity, through cultural traditions, that some of those [assimilation] effects will seem to be a bit stronger to you and then the southern dependency needs to be more so. And, so I think the emotional health of people through [land-based] activity and through [country] food and the ability to produce something through their activity will be negatively impacted if the climate change affects us severely … I think if you take away those [land-based] activities and people feel less capable, less able to provide, and less healthy about themselves then those [assimilation] impacts will either come more to the forefront and have to be dealt with, or they [assimilation impacts] may just be built upon.

Moreover, experiencing stress and anxiety about safety and new hazards on the land as a result of the observed changes in climate was described by many government interviewees and community members. Interview and PV participants described being “frustrated,” “dissatisfied,” “distressed,” and “unsettled,” and expressed feelings of “worry,” “anxiety,” “disappointment,” and “sadness” about the climatic variability observed in the previous year; a sentiment which is supported by previous research [8, 57]. Similar to these findings, Nain community members reported worry and stress for themselves or for family members travelling on the land related to recent changes in ice conditions, as well as reduced predictability of ice and weather conditions [70]. As one participant explained,

Every conversation was around the ice was thin, it was unsafe to go, then they added some worry to that because people were still craving to get out on their skidoos on thin ice, with people going through the ice, and then there were family members that was off and didn’t come back. So, a lot of extra anxiety and disappointment, and unfulfilled needs…it [the weather] was definitely different than it was expected here normally.

In the BPS survey, the odds of reporting feeling angry, scared, and frustrated by changes in the environment were higher for women compared to men (Table 2). Past research has primarily focused on Inuit men’s climate change observations and perceived impacts, resulting in a more limited understanding of how climatic change impacts are experienced by women [91, 92]. Thus, further in-depth investigation of the gendered impacts of climatic change on Inuit health is warranted.

#### Social Cohesion: “a common denominator of outdoor-based activities”

Similar to other Inuit communities [93], participants explained that interpersonal “relationships are really important and everybody is really connected” in the Nunatsiavut region. All interviewees and PV participants reported that social connections were an important aspect of Inuit culture, and an important aspect of individual and community health. Participants described the land as an important platform for people to connect, and essential to strengthen and support kinships and social support networks. As one Inuit Elder explained,

My mom and I, God rest her soul, used to have our absolute best times berry picking… we talked about things we've never talked about before because it's very relaxed, beautiful day, and you’re out. Then you got your picnic lunch, and you're just picking berries and you're chattin’, she might be over there and I'm over there and everything was sa.., it’s safe, it’s a safe place to, even air things that are never talked about, is when you're out berry picking. It's wonderful. And you're walking and you’re bending and, it's just good for your soul, it’s good for everything.

Participants explained that observed changes either already have, or had the potential to impact their ability to access the land and develop and reinforce these relationships, which has important health implications (Fig. 4, Table 2). An interviewee explained that during the past winter “people couldn’t go out and hunt, they couldn't go in between communities, which is a critical piece of the socialization of Inuit. It was really challenging this year to be able to do that.” One community member worried about implications of continued climatic change on community cohesion,

I think that community health is based on cohesiveness, and the cohesiveness doesn’t have to be physical. It doesn’t mean that you visit all the time, which is great as well. It doesn’t mean that you have to be seeing people all the time. But, it usually means there’s some camaraderie in [land-based] activity… there’s a cohesiveness that needs to continue, and if that cohesiveness is usually on a common denominator of outdoor-based activities, cultural based activities, it’s sort of somewhat fragmented [because of climate change]. The cohesiveness that now bonds the community could be jeopardized because what else are you bonding on?

#### Western and Indigenous Education and Knowledge: “Knowledge is still passed on; the relevance of it all becomes a question”

Participants identified two types of education in Nunatsiavut: (1) formal institutionalized training and (2) informal or traditional ways of learning. Challenges facing both types of education were described, but changes and challenges to traditional ways of learning were more often the focus of discussion (Fig. 2f). Government interviewees and PV participants believed that the land was fundamental to informal traditional learning. Participants explained that while there were a variety of factors impacting informal education (e.g. wage economy, Elders passing on, formal education; c.f. Pearce et al. 2011), the observed changes in climate were clearly identified by participants as a stressor on informal education in two ways: first, it was reported that decreased access to the land was resulting in fewer opportunities to orally pass on traditional knowledge; second, the relevance of the traditional knowledge was questioned by some interviewees. As one community member explained, “it’s different because when you’re growing up, you’re taught where to go and where not to go ‘cause there’s bad parts of the ice. Ah, it seems like today or this past winter, you had [bad] places that are existing where they shouldn’t be, where they weren’t before.” As such, participants believed “that knowledge is still passed on; the relevance of it all becomes a question.” Nonetheless, PV participants agreed that some of the fundamental or “core” knowledge, especially related to Inuit values, will always remain applicable, regardless of climatic change. For instance, one community member explained, “it’s almost always the same message… the general part of it is still there you just have to apply it differently now ‘cause things have changed.” Indeed, research on traditional knowledge systems and climatic change conducted with Inuit hunters in Nunavut and the Northwest Territories describes an evolving nature of traditional knowledge, in which new knowledge heuristics are evolving in-light of observed changes [59, 63, 94].

#### Housing: “Couch-surfing, homelessness, and lack of sufficient housing”

Participants reported that, similar to other Inuit regions [95, 96], “housing is a huge issue” in Nunatsiavut. Government interviewees reported several problems with the quantity and quality of houses, resulting in “very limited housing,” “lack of adequate housing,” “homelessness,” and “over-crowding.” Participants believed that climatic change was worsening the existing housing challenges in the region. For instance, participants reported that warming temperatures have introduced mould problems to the region. Government interviewees were particularly concerned about increasing mould in houses, especially considering the already high levels of respiratory illness in Inuit communities [97, 98]. As one interviewee explained, “there’s a lot more mould, and you're going to get that too in a milder climate. You’re going to have more rain, you're going to have more mould. And of course mould has direct impact on lung health.” Furthermore, a participant explained, “the association between climate change and the increase in mould in homes [is concerning], because usually the frost kills everything, nothing’s going to survive, right? You don't have that now.” Other research, however, has indicated generally low humidity levels in homes, and consequently low levels of mould in Inuit communities [97, 98]. Therefore, mould and related respiratory illness could become an emerging climate-related health concern in the region.

Participants were also concerned about damage to infrastructure from erosion, shifting grounds, and damage from heavy storms. Participants worried that in the future these changes might result in further damage to infrastructure and housing that was already in need of repair and further worsen over-crowding and mould problems; a concern shared in other Inuit communities [99].

#### Healthcare Services: “Huge effects on planning”

While it was not discussed by community members, all government interviewees described several challenges in healthcare provision in the north: “health care delivery for rural and remote areas is often very complex and very difficult.” These challenges are similar to those reported in many Indigenous communities in Canada (e.g. high staff turn-over, geographic remoteness, small population sizes, and increased travel costs) [100, 101], resulting in Northern regions having the highest per-capita health care expenditures in Canada [100].

Government interviewees linked several aspects of healthcare provision to weather conditions. As one healthcare worker explained, “there’s no doubt about it that weather [is important], it’s a key factor in everything we do every hour of the day.” Interviewees most commonly identified transportation as a climate-sensitive challenge in healthcare provision. Similar to most remote Inuit communities [101], community members travel via airplane outside of the community to a southern health centre for many healthcare services, and healthcare staff often travel via airplane between communities to provide care. As such, in Nunatsiavut, air transport places a large economic burden on the healthcare system [102]. Participants explained that the increase in extreme weather conditions (e.g. winds, milder temperatures resulting in freezing rain, snow storms) interrupted air-travel, which impacted the ability of community members to leave the community for treatment, evacuate medical emergencies, transport medical samples, and prevented healthcare workers from travelling to communities that were in need. Government interviewees explained that these increased travel interruptions were placing further stress on the healthcare system. For instance, one government interviewee explained, “if we have a mild winter like we did this year and the weather is down [bad] for long periods of time, it drives our budgets through the roof.” Another government interviewee explained,

There is not as much snow as we normally get, and it definitely had an effect–but being able to get staff into the community–or staff in the community can’t get out of the community, patients can’t get to the hospital for their appointment if they need to do that. People that came out for appointments can’t get back into the community–so it is huge–it is huge and those [travel interruptions] were not normal. You know, 11, 12 days at a time when the planes can’t get into the community is not normal for here.

Government workers also explained how climatic variability was impacting the ability to plan and implement health programming. Several examples were provided to illustrate how unusual weather was “interrupting plans”, causing “major disruptions” for healthcare programming that had “huge implications” and “huge effects on planning”, especially for land-based programs (e.g. mental wellness and additions programs). Participants also reported that increasing extreme weather conditions lead to communication outages along the coast,

You have these freak periods of heavy snow that knock down the radio tower that knock down the telecommunications, so then people were literally back in the old days… but that is how climate change can affect so many layers of remote living. Right from using your ATM card to being able to fly out because someone is having a heart attack. It’s all related.

As such, climate-related adaptation planning in public and primary health programming, policies, procedures, and regulations was identified by participants as important moving forward, which is supported in the literature [10].

### Climate-sensitive Health Priorities for the Nunatsiavut Region

**Fig. 5:**
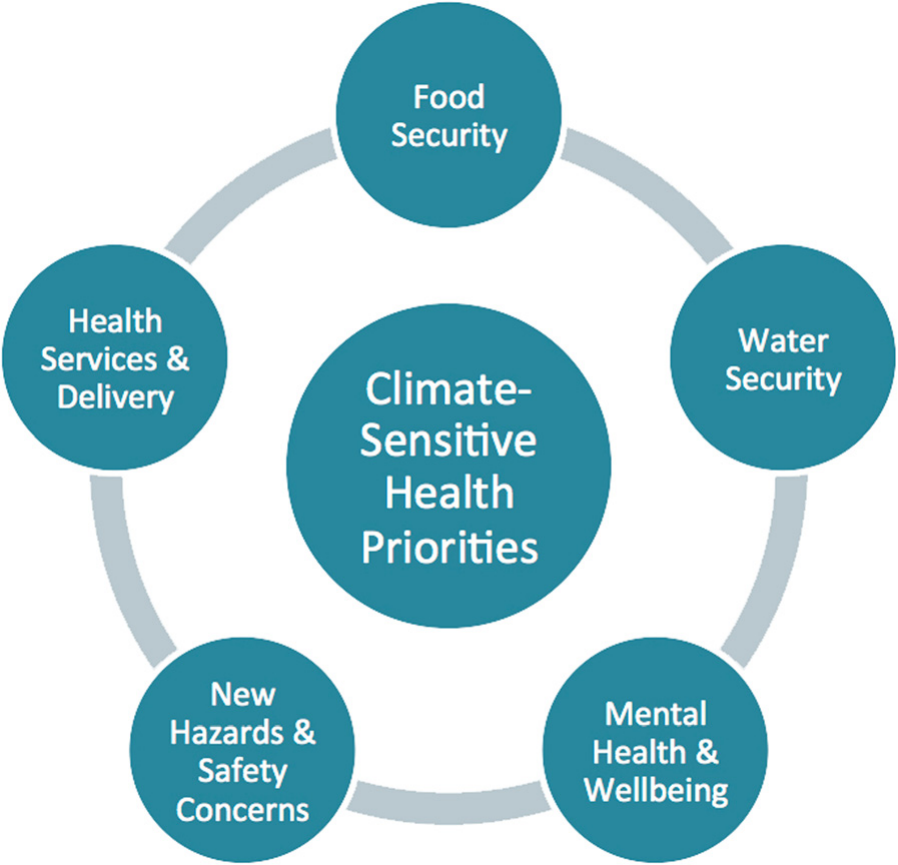
Top climate-sensitive health priorities identified by participants in Nunatsiavut, Canada

After participants described and discussed climate-health relationships, interviewees were then asked to identify and rank what they perceived to be the most important climate-sensitive health impacts in the present and in the near-future. Government employees and PV participants identified the following as the top climate-sensitive health priorities in the Nunatsiavut region: food security, water safety and security, mental and wellbeing, new hazards and safety concerns on the land, and health services and delivery (Fig. 5). Participants, however, emphasized that these top five priorities should not be ranked; as one Inuit Elder described, these priorities should be presented as a “circular model. They tie in and they impact each other, and their position on the circle shifts from time to time, but they are interrelated.”

### Study limitations

Several limitations of this study should be noted. First, caution should be used in extrapolating these results beyond Nunatsiavut, since the impacts of changing climatic conditions are often unequally distributed and differentially experienced at individual, family, and regional scales. Rather, the goal of this study was to characterize climate-sensitive health priorities for Nunatsiavut, and pilot a participatory process of characterizing climate-related health priorities that could be used in other regions. Second, while data collection in Rigolet provided a community perspective to the research and contextualized the insights provided by regional stakeholders, we acknowledge that the climatic change observations and priorities likely vary for each Nunatsiavut community. While participants described changes in weather observed over their lifetime, most examples that participants provided to illustrate the impacts of these climatic changes were from their immediate experiences in the last 5–10 years. Thus, further research is required to differentiate the impacts of long-term climatic change from year-to-year climatic variability. Finally, these types of assessment are intended to be an ongoing assessment and action process, not a single assessment of risks and interventions conducted at one point in time [22]. As such, the priorities identified in this research should be re-visited, modified, and updated moving forward to enhance health responses to climate change.

## Conclusions

This study used a participatory approach to identify and describe the climate-sensitive health priorities in Nunatsiavut, Labrador, Canada. Participants described the environment as being inextricably linked to several determinants of Inuit health, which increases sensitivity to climatic change related impacts on health. This view coincides with EcoHealth concepts, which posits that human health and wellbeing is a function of complex social and ecological interactions [103]. EcoHealth concepts not only explicitly link environmental and social determinants of health, but also provide a framework or “a mindset that orients a process of inquiry that is meant to lead to some action or change” (Charron 2012, pg. 32). Therefore, our results suggest that EcoHealth approaches could be mobilised to support health-related adaptations to climatic change in Inuit regions. For instance, past research has recommended enhanced, integrated, and community-led surveillance, warning systems, hazard epidemiology, harvester support, co-management of wildlife resources, land-skills training, food system enhancement, infrastructure protection, and emergency management, among others to respond to climate change impacts in the Canadian Arctic [12, 104–105]. To realize these benefits and plan for climate change impacts, adaptation policies, programs, and interventions need to build on and integrate EcoHealth principles of systems thinking, transdisciplinarity, community participation, social and gender equity, and knowledge-to-action. Integrating these principals into policy development and program planning has been proposed [106–109] and successfully used [7, 110–114] in other environmental health programs, and our research results suggest it could also be useful in a climate-health context.

Importantly, these priorities were identified through a participatory process that engaged health professionals, government decision makers, and community members. This process underpinned the local and cultural relevance and accuracy of the climate-sensitive health priorities identified, and places the priorities within an appropriate societal, cultural, environmental, political, and economic context for the Nunatsiavut region. Furthermore, engaging local stakeholders in the priority identification process is commonly recommended in climate change policy literature as it enhances policy uptake and applicability, increases legitimacy of priority areas, enables integration of Inuit knowledge, increases community representation in decision-making, and increases local capacity to respond to climate-related stressors [12, 22, 104–105, 115]. As such, the five priority areas (Fig. 5) identified by local stakeholders provides a reference point for local, regional, and provincial decision makers to assess and enhance existing, or create new climate change strategies, policies, and programing. For instance, in reviewing the Province of Newfoundland and Labrador climate change action plans and initiatives [116], some of the climate-sensitive health priorities identified in this study are being acted on (e.g. an aim to improve infectious disease surveillance, which, if implemented, could help reduce waterborne disease); however, other climate-sensitive health priorities do not have specific response plans, actions, or programming (e.g. mental health). By identifying and prioritizing climate-sensitive health priorities, this research provides the foundation and focus for future health-related climate change assessments, action plans, and adaptation plans in the region.

In summary, this study provided baseline understanding of the climate-sensitive health issues affecting Labrador Inuit, which included the following as top health priorities: food security, water security, mental health and wellbeing, new hazards and safety concerns, and health services and delivery. These climate-sensitive health issues were experienced through complex inter-relationships between environmental and social determinants of Inuit health. This work, in turn, has guided the development of a 5-year research program focusing on Indigenous health and climate change, including research focusing in greater depth on these priority areas in Rigolet. Several climate-sensitive health outcomes have been identified at national or global scales, and many of the climate-sensitive health priorities described in our study are not only Northern issues [18, 20, 41, 76, 117], but international issues [4, 27, 118–123]. Nonetheless, how climate change impacts are felt and responded to most often varies at local scales and is dependent on location-specific socioeconomic, cultural, and biophysical factors [10]. As such, local assessments and prioritization of current and future climate change impacts on health are required [10, 22, 25].
